# 

**Published:** 1904-01

**Authors:** 


					﻿ATLANTA
JOURNAL-RECORD
iDB MEDICINE.
SUBSCRIPTION $1 OO.
PUBLISHED MONTHLY
Successor to Atlanta nedfcal and Surgical Journal, Established 1855,
and Southern fledical Record, Established 1870.
Vol. V.	Atlanta, Ga., January, 1904.	No. 10.
				

